# The Role of Alternative Electron Pathways for Effectiveness of Photosynthetic Performance of *Arabidopsis thaliana*, Wt and *Lut2*, under Low Temperature and High Light Intensity

**DOI:** 10.3390/plants11172318

**Published:** 2022-09-04

**Authors:** Antoaneta V. Popova, Martin Stefanov, Alexander G. Ivanov, Maya Velitchkova

**Affiliations:** 1 Institute of Biophysics and Biomedical Engineering, Bulgarian Academy of Sciences, Acad. G. Bonchev Str., Bl. 21, 1113 Sofia, Bulgaria; 2 Department of Biology, University of Western Ontario, 1151 Richmond Str. N., London, ON N6A 5B7, Canada

**Keywords:** alternative electron flows, combined abiotic stress, carotenoid mutant, cyclic electron transport, PGR5, photoprotection, PTOX

## Abstract

A recent investigation has suggested that the enhanced capacity for PSI-dependent cyclic electron flow (CEF) and PSI-dependent energy quenching that is related to chloroplast structural changes may explain the lower susceptibility of *lut2* to combined stresses—a low temperature and a high light intensity. The possible involvement of alternative electron transport pathways, proton gradient regulator 5 (PGR5)-dependent CEF and plastid terminal oxidase (PTOX)-mediated electron transfer to oxygen in the response of *Arabidopsis* plants—wild type (wt) and *lut2*—to treatment with these two stressors was assessed by using specific electron transport inhibitors. Re-reduction kinetics of P_700_^+^ indicated that the capacity for CEF was higher in *lut2* when this was compared to wt. Exposure of wt plants to the stress conditions caused increased CEF and was accompanied by a substantial raise in PGR5 and PTOX quantities. In contrast, both PGR5 and PTOX levels decreased under the same stress conditions in *lut2*, and inhibiting PGR5-dependent pathway by AntA did not exhibit any significant effects on CEF during the stress treatment and recovery period. Electron microscopy observations demonstrated that under control conditions the degree of grana stacking was much lower in *lut2*, and it almost disappeared under the combined stresses, compared to wt. The role of differential responses of alternative electron transport pathways in the acclimation to the stress conditions that are studied is discussed.

## 1. Introduction

Plants are frequently facing adverse environmental stress conditions such as extreme temperatures, differences in optimal light illumination, high salinity conditions, dehydration, etc., that quite often act in a simultaneous manner and seriously affect the plant’s development and distribution. The effective functioning of photosynthetic reactions is largely dependent not only on the development conditions, but as well on the ability of plants to maintain a balance between the amount of absorbed sunlight and a capacity for its utilization through biochemical reactions. The exposure of plants to the simultaneous actions of increased light intensity and low temperature can lead to the over-reduction of the electron carriers of the photosynthetic electron transport chain [1,2], to oxidative stress and to the photoinhibition of photosystem II (PSII) [3,4] and photosystem I (PSI) [5,6]. 

Higher plants have evolved a number of mechanisms that effectively dissipate the excess amounts of absorbed light for maintaining the balance between the absorbed energy and the biochemical sinks. It is general considered that the main photoprotective mechanism is the non-photochemical quenching of the excited chlorophyll states (NPQ), operating in the light-harvesting complex of the PSII (LHCII) that is dependent on the functioning of xanthophyll cycle and triggered by the buildup of the proton gradient across the thylakoid membrane [7,8]. Other mechanisms of balancing the absorbed and utilized energy are also reported; by adjusting the absorption cross section of PSII, the transfer of energy from PSII to PSI [1,9], quenching in the reaction center of PSII via a charge recombination [10] or by constitutive energy dissipation (Φ(NO)) [11]. Under the conditions of the over-excitation of PSII, a cyclic electron flow (CEF) around PSI is recognized as a mechanism, protecting PSII and PSI against photoinhibition and providing additional ATP for improving the ratio of ATP/NADPH that is needed for metabolic reactions at the acceptor side of PSI [12,13]. The functioning of CEF is essential for the increase of the proton gradient across the thylakoid membrane and for facilitating the NPQ and the protection of PSII from oxidative stress [14,15]. 

Under physiological conditions, the main electron transport pathway during photosynthesis is the linear electron transport chain (LET), but under stress conditions, in cyanobacteria, green alga and higher plants, when the CO_2_ fixation and the generation of ATP are limited, alternative electron transport flows around PSI are recognized as essential for the generation of the proton motive force, which is used for the induction of NPQ and photosynthetic control at the level of the cytochrome b_6_f (Cyt b_6_f) [15,16,17,18,19]. Electrons from NADPH or reduced ferredoxin are recycled around PSI and directed back to the plastoquinone pool (PQ), and the Cyt b_6_f that is coupled to the buildup of ΔpH is used for the synthesis of ATP [20,21] or for providing an efficient photoprotection of PSI and PSII at high light conditions [16,17,22,23,24,25,26]. Diverting the excess electrons from the acceptor side of PSI decreases the formation of the extra reactive oxygen species (ROS), thus diminishing the oxidative stress [15,22,27]. 

It is considered that, in angiosperms, CEF is realized by two pathways; one is sensitive to antimycin A (AntA) that involves the activity of ferredoxin plastoquinone (Fd-PQ) reductase and proton gradient regulation 5 (PGR5-mediated), and the second is related to NAD(P)H dehydrogenase-like protein (NDH-1 complex). Both pathways (PGR5- and NDH-1-dependent) of recycling electrons around PSI act in a complimentary manner [22], and the activity of each one is dependent on the respective plant species and/or environmental conditions [28]. The AntA-sensitive (PGR5-dependent) CEF is suggested to be the main CEF pathway for C3 plants, especially under stress conditions [29], but the contribution of the minor CEF pathway (NDH-1-dependent) also plays an important role [30,31,32].

The AntA-sensitive pathway of CEF [33] requires the operation of two proteins—proton gradient regulation 5 (PGR5) [16] and PGR like 1 (PGRL1) protein [34]. At high light conditions, NPQ was strongly suppressed in the *pgr5* mutant of *Arabidopsis*, indicating that the buildup of the membrane proton gradient was significantly impaired and that the PGR5/PGRL1-dependent pathway of electron recycling around PSI is essential for the dissipating of the excess absorbed light and protection of PSI against photodamage [16,22]. 

Nevertheless, the contribution of the NDH-1-dependent pathway to the overall CEF is considered to be minor but its involvement in the generation of the proton motive force under abiotic stress can be significant [20,35,36]. 

Another enzyme that is related to CEF around PSI is the plastid terminal oxidase (PTOX), which represents a plastoquinone-oxygen oxidoreductase (diiron-containing protein). PTOX is involved in the process of chlororespiration, which is expressed in accepting electrons from PQH_2_ for the reduction of O_2_ to water in chloroplasts that are in the dark. Despite this, dark chlororespiration is not directly related to the PSI-mediated alternative electron transport, as it shares electrons with PSI under light [15,37]. In tobacco plants with over-expressed PTOX from *Arabidopsis thaliana*, the activity of CEF can be controlled at the level of PQ by PTOX [37] and it does not have a significant impact on the protection of the photosynthetic apparatus [38]. Under optimal growth conditions, the amount of PTOX in wt of *Arabidopsis* is very low, comprising only 1% of the other electron transport components [37]. However, under stress conditions, its amount is increased, reaching 30% of that of the photosynthetic electron carriers [11,39,40,41], thus pointing out that PTOX can be involved in the plant stress response. One of the most reviewed roles of PTOX in chloroplasts is its role to serve as a safety valve for the utilization of excess electrons under stress conditions that can lead to photo-oxidative stress and photoinhibition [42,43,44]. 

Carotenoids are an essential constituent of pigment-protein complexes that perform multiple functions. The oxygenated representatives of carotenoids, xanthophylls, are bound to the proteins of the LHCs of PSII and PSI, and each of them is characterized with an unique xanthophyll composition [7,45]. The role of xanthophylls for the formation and stabilization of the trimeric structure of LHCII in cyanobacteria and higher plants, and their participation in the absorbing and dissipation of excess absorbed light [46] are well recognized. For unravelling the specific role of xanthophylls and the specificity of their binding sides to light-harvesting proteins, different xanthophyll mutants of *Arabidopsis thaliana* are investigated. One of the most frequently studied *A. thaliana* xanthophyll mutant is *lut2*, that, due to the inactivation of lycopene ɛ-cyclase gene, does not synthesize lutein, that is the most abundant xanthophyll in higher plants [47]. The absence of lutein in *lut1* and *lut2* mutants leads to a compromised trimeric organization of the LHCII complexes, which negatively impacts the efficiency of NPQ [48]. In the absence of lutein, the quenching of ROS is less effective, but the extent of photoinhibition and degradation of LHCII proteins are higher [49,50]. Recent studies have suggested that an enhanced capacity for PSI-dependent CEF may ameliorate the negative effect(s) of the combined stress conditions of a low temperature and a high light intensity in *lut2* mutant [51,52].

The objective of the present study was to evaluate whether and to what extent the major alternative CEF, the PGR5-dependent pathway, is involved in the response and recovery of *Arabidopsis thaliana* plants—wt and *lut2*—to simultaneous treatment with two stress factors: a low temperature and a high light intensity, followed by a recovery period at control conditions. The importance of PTOX as a potential electron sink under the application of a double stress treatment was evaluated in parallel. Two specific electron transport inhibitors were applied—AntA for PGR5 and octyl gallate (OG) — for PTOX.

## 2. Results

### 2.1. Effect of Specific Electron Transport Inhibitors on PSII Activity in Leaf Discs of wt and lut2 Plants That Were Treated with Two Stress Factors and Recovered 

The contribution of the alternative electron transport pathways to the photosynthetic response of PSII in wt and *lut2 Arabidopsis thaliana* mutants to the combined treatment of low temperature and high light intensity for different time periods was estimated by using specific electron transport inhibitors - AntA for PGR5-dependent CEF and OG for PTOX-dependent electron transfer to oxygen.

**Figure 1:**
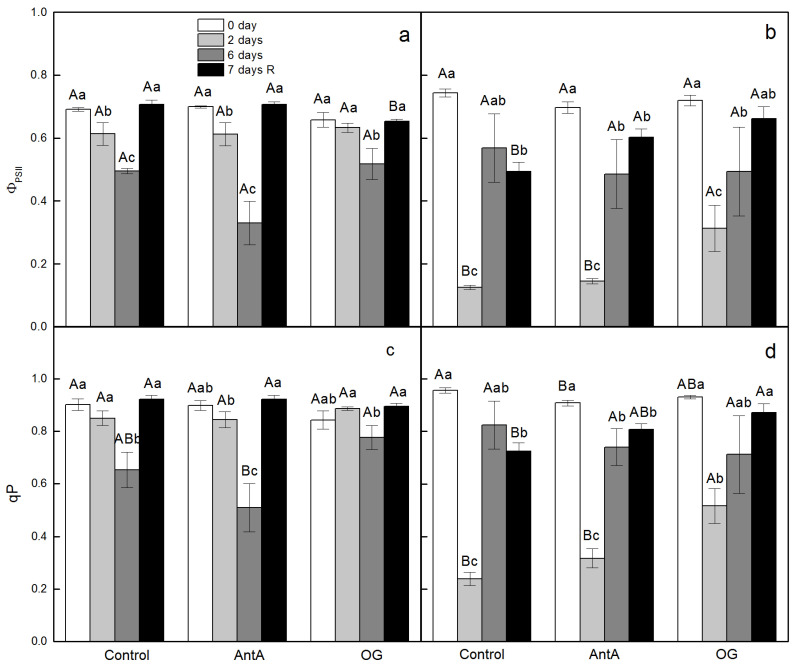
Effect of specific electron transport inhibitors on the effective quantum yield of photochemical energy conversion of PSII (Φ_PSII_) (**a**,**b**), and on the coefficient of photochemical quenching (qP) (**c**,**d**) of leaf discs from *Arabidopsis thaliana* wt (**a**,**c**) and *lut2* (**b**,**d**) plants before start of treatment (0 day), after treatment of plants for 2 or 6 days with low temperature and high light intensity, and after a recovery period at normal conditions (7 days R). Leaf discs were floated either on distilled water (Control) or in the presence of two electron inhibitors - 10 µM antimycin A (AntA) for protein gradient regulator 5 (PGR5) or 20 µM octyl gallate (OG) for plastid terminal oxidase (PTOX). Results presented are mean values ± standard error (SE) from three parallel samples of two independent experiments (*n* = 6). Different letters indicate significant differences for the respective parameters at *p* < 0.05 (uppercase for the different treatments (Control, AntA and OG) on a certain day; lowercase for the certain treatment at different time periods (0 day, 2 days, 6 days and 7 days R)).

For wt discs, which were infiltrated for 2 h with distilled water (Control), a decrease of the quantum efficiency of the electron transport through PSII (Φ_PSII_) was observed after 6 days of treatment with two stress factors, while for *lut2* discs the decline of this parameter was much stronger expressed and it occurred on the second day of the treatment (Figure 1a,b). After a recovery period in control conditions, the quantum yield of PSII was completely recovered in the wt discs, but for the mutant, the value was still low, in comparison with that of the non-treated plants. The presence of both inhibitors, AntA and OG, did not significantly alter the values of Φ_PSII_ in the non-treated plants of wt and *lut2*. In the presence of AntA, Φ_PSII_ decreased in the wt on the 6th day of the treatment, in comparison with the discs without inhibitors. For the *lut2* discs, AntA expressed a little, if any, effect on the value of Φ_PSII_ on the second day of the treatment. The application of OG did not affect the Φ_PSII_ in the wt discs on the 6th day of the treatment, while the value of Φ_PSII_ in the *lut2* discs on the second day was two times higher in comparison with that which was infiltrated with water or in the presence of AntA (Figure 1a,b). Similar alterations in the wt and *lut2* discs were detected for the coefficient of photochemical quenching (q_P_) (Figure 1c,d).

**Figure 2:**
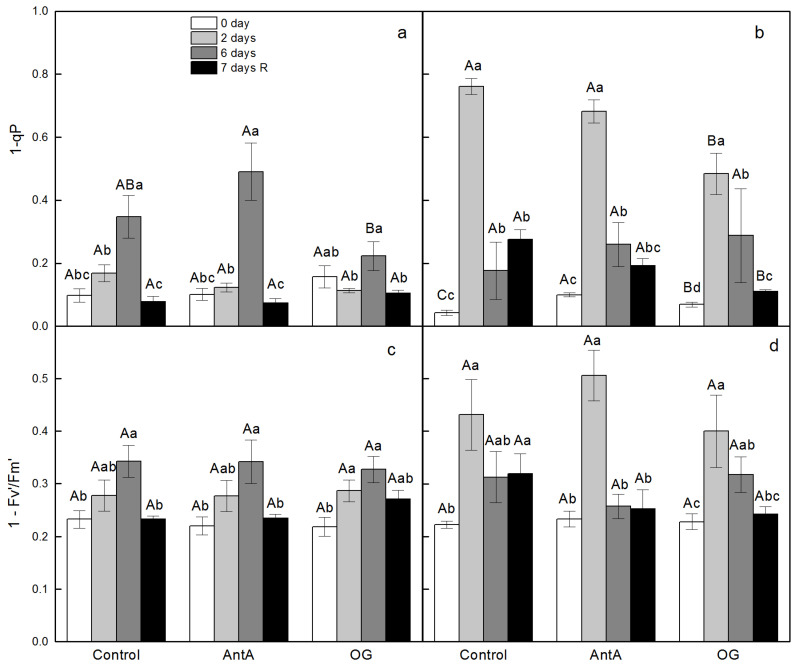
Effect of specific electron transport inhibitors, antimycin A (AntA) for protein gradient regulator 5 (PGR5) and octyl gallate (OG) for plastid terminal oxidase (PTOX), on the excitation pressure of PSII (1-q_P_, q_P_—coefficient of photochemical quenching) (**a**,**b**) and on the proportion of light energy absorbed and dissipated in the antenna of PSII (1-F_v_´/F_m_´, where F_v_′ is the variable fluorescence in light acclimated state equal to F_m_′-F_o_′, where F_o_′ and F_m_′ are the minimal and maximal chlorophyll fluorescence levels in light acclimated state, respectively) (**c**,**d**) in leaf discs of *Arabidopsis thaliana*, wt (**a**,**c**) and *lut2* (**b**,**d**), that were floated for 2 h either on distilled water (Control) or in the presence of inhibitors. Leaf discs were taken either from plants before the start of every experiment (0 day), after 2 or 6 days of treatment with two stress stimuli or after the recovery period (7 days R). The concentration of inhibitors in distilled water was—10 µM AntA or 20 µM OG. Mean values ± standard error (SE) were calculated from three parallel samples of two independent experiments (*n* = 6). Different letters indicate significant differences for the respective parameters at *p* < 0.05 (uppercase for the different treatments (Control, AntA and OG) on a certain day; lowercase for the certain treatment at a different time periods (0 day, 2 days, 6 days and 7 days R)).

In Control leaf discs, the values of the excitation pressure on PSII (1 *-* q_P_*)* were elevated in the wt and the *lut2* over the course of treatment with two stress factors, reaching values of 0.35 for wt on the 6th day of the treatment and 0.75 for *lut2* on the second day (Figure 2a,b). During the recovery period, the values of 1-q_P_ decreased significantly for both types of discs, but they remained higher for *lut2* in comparison with the non-treated plants. In the presence of AntA or OG, the values of 1-q_P_ on the second day of the treatment in *lut2* were lower than those that were infiltrated without inhibitors. For the wt discs, the presence of AntA increased 1-q_P_ on the 6th day of the treatment, while after the infiltration with OG, the value was much lower than those that were floating on water, for wt and *lut2* (Figure 2a,b). Similar to the excitation pressure, the relative proportion of the energy that was absorbed and dissipated as heat in the PSII antennae (1-F_v_′/F_m_′ where F_v_′ is the variable fluorescence in the light acclimated state equal to F_m_′-F_o_′, F_o_′ and F_m_′ are the minimal and maximal chlorophyll fluorescence levels in the light acclimated state, respectively) increased in the wt discs on the 6th day of treatment and on the second day in the *lut2* discs. (Figure 2c,d).

### 2.2. Effect of Specific Electron Transport Inhibitors on the Redox State of PSI after Treatment of wt and lut2 Plants with Two Stress Factors

**Figure 3:**
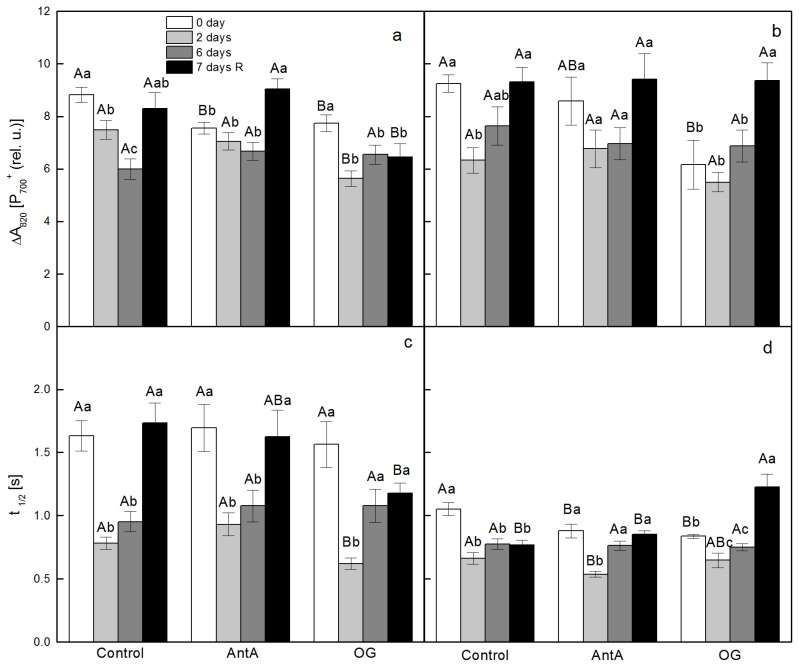
Alterations in far-red (FR) induced oxidation of reaction center of PSI (P_700_) to (P_700_^+^), as estimated by the absorbance change at 820 nm (ΔA_820_) (**a**,**b**) and the capacity for cyclic electron transport through PSI (CEF), as assessed by determination of the half time (t_1/2_) of the dark re-reduction of P_700_^+^ after switching off the FR light (**c**,**d**). Leaf discs of *Arabidopsis thaliana*, wt (**a**,**c**) and *lut2* (**b**,**d**), were floated for 2 h either on distilled water (Control) or on distilled water containing 10 µM antimycin A (AntA) or 20 µM octyl gallate (OG). Leaf discs were cut from non-treated plants (before start of every experiment, 0 day), after 2 or 6 days of treatment with low temperature and high light intensity or after a recovery period (7 days R). Results presented are mean values ± standard error (SE) from three parallel samples of two independent experiments (*n* = 6). Different letters indicate significant differences for the respective parameters at *p* < 0.05 (uppercase for the different treatments (Control, AntA and OG) on a certain day; lowercase for the certain treatment at a different time periods (0 day, 2 days, 6 days and 7 days R)).

The alterations in PSI photo-oxidation (reaction center of PSI (P_700_^+^)) were evaluated by following the far red (FR)-induced absorbance changes at 820 nm (ΔA_820_) that correlates with the degree of P_700_ oxidation to P_700_^+^. Similar to changes in the electron transport through PSII, as determined by Φ_PSII_ and q_P_ (Figure 1), the lower degree of oxidation of P_700_ in the wt discs that were floating on distilled water (Control) was detected on the 6th day of treatment and in *lut2* discs, this was after 2 days (Figure 3a,b). The oxidation extent of P_700_ in the wt discs from the non-treated plants decreased by around 15% in the presence of both inhibitors. OG exhibited a stronger effect on the decline of P_700_^+^ in the leaf discs from the *lut2*, non-treated plants. The effect of AntA was more evident in the leaf discs from the treated, *lut2* plants, while OG was more effective in the wt discs under the same conditions. 

Figure 3c,d presents the values of the half time (t_1/2_) of the re-reduction of P_700_^+^, as a measure of CEF around PSI. The detected t_1/2_ in the wt discs from the non-treated plants, which were infiltrated only with water (Control), was 1.63 s and did not change considerably in the presence of AntA and OG. The t_1/2_ in the *lut2* from the non-treated plants, which were infiltrated with water, showed a lower value of 1.05 s. The presence of both inhibitors accelerated the CEF after the treatment, with OG being more effective in the wt discs, and AntA realizing a stronger effect in the *lut2* discs, with nearly 20%. The presence of both inhibitors accelerated the CEF in the *lut2* discs from the non-treated plants, with nearly 20%. For the discs of the treated plants, AntA and OG were more effective in accelerating the rate of CEF in the wt than they were in the *lut2* plants.

### 2.3. Intersystem Electron Pool Size and PQ Pool Reduction

**Figure 4:**
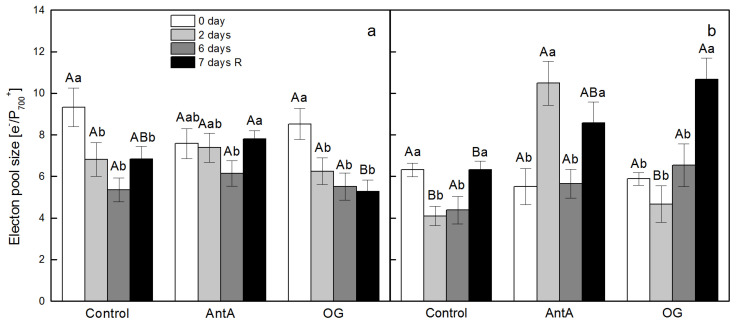
Effects of electron transport inhibitors - 10 µM antimycin A (AntA) or 20 µM octyl gallate (OG) - on the size of the intersystem pool of electrons that can be donated to P_700_^+^ in leaf discs of *Arabidopsis thaliana*, wt (**a**) and *lut2* (**b**). Leaf discs were infiltrated for 2 h on distilled water (Control) or on distilled water containing AntA or OG. Leaf discs were cut from non-treated plants, after 2 or 6 days of treatment with low temperature and high light intensity or after a recovery period (7 days R). Results presented are mean values ± standard error (SE) from three parallel samples of two independent experiments (*n* = 6). Different letters indicate significant differences for the respective parameters at *p* < 0.05 (uppercase for the different treatments (Control, AntA and OG) on a certain day; lowercase for the certain treatment at a different time periods (0 day, 2 days, 6 days and 7 days R).

The registered intersystem electron pool size in the leaf discs from the *lut2*, non-treated plants, which were floated on water (Control), was around 30% lower than that in the wt discs (Figure 4a,b). In the wt discs from the treated plants and those which floated on water (Control), a gradual decrease in the pool size was detected with the increase of the duration of the stress treatment—2 and 6 days (Figure 4a), while in *lut2* discs the decline was similar for the 2nd and 6th day of the treatment—of around 30% (Figure 4b). The application of both inhibitors resulted in a similar decline in the electron pool size in the discs from the non-treated plants of wt and *lut2*, with 13% and 7% for AntA and OG, respectively. The most prominent difference between the wt and *lut2* discs was found in the discs of the treated plants in the presence of AntA; for the wt discs, the decline was by 20% after 6 days of treatment (Figure 4a), but for *lut2* discs on the second day of treatment, the electron pool size was twice higher than in the respective non-treated plants when AntA was applied (Figure 4b).

**Figure 5:**
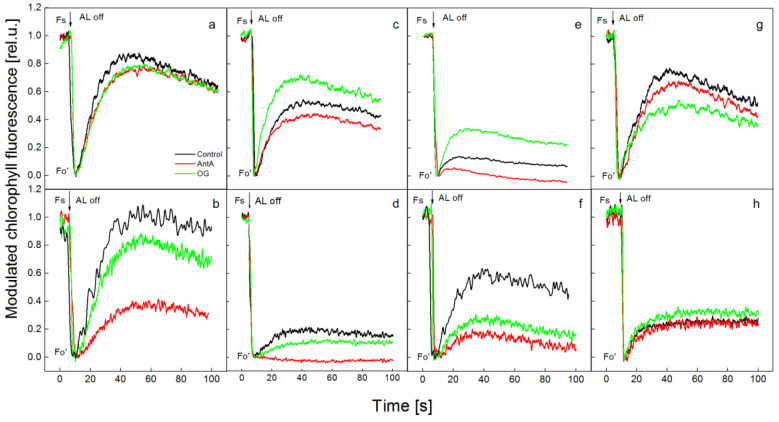
Post-illumination increase of minimal fluorescence levels in light acclimated state (F_o_´) transients in leaf discs from *Arabidopsis thaliana*, wt (**a**,**c**,**e**,**g**) and *lut2* (**b**,**d**,**f**,**h**), in non-treated discs (**a**,**b**), those treated for 2 (**c**,**d**) or 6 (**e**,**f**) days with two stress factors and after a recovery period at normal conditions for 7 days (7 days R) (**g**,**h**). Leaf discs were floated on distilled water (Control), indicated by the black traces, in the presence of antimycin A (AntA) (red traces) or octyl gallate (OG) (green traces). The traces in every panel were normalized between one and zero for better visualization. Every transient was the average from three parallel records in two independent experiments (*n* = 6). AL - Actinic light. F_s_ and F_o_′—steady state fluorescence level and minimal fluorescence level in light acclimated state, respectively.

Figure 5 includes the post-illumination increases of F_o_′ after switching off actinic light (AL) of the leaf discs of wt (Figure 5a,c,e,g) and of *lut2* (Figure 5b,d,f,h) which were floated on water for 2 h (Control) or in the presence of inhibitors before start of treatment (a,b), after the treatment for 2 (c,d) or 6 days (e,f) and after a recovery period (g,h) for the plants. The transient increase of the F_o_′ in the discs from the non-treated plants, an indicator of a non-photochemical reduction in the PQ pool, represents 89% of the increase from the F_o_′ to the steady state fluorescence level (F_s_) for wt, while for the *lut2* discs, this increase was slightly higher than the F_s_ (a,b). After 2 and 6 days of the treatment and in the absence of inhibitors, the transients for the wt showed a lower level of reduction, being the lowest on the 6th day of the treatment, representing 13% of the increase of F_o_′ to F_s_ (black traces in Figure 5a,c,e,g). 

In the *lut2* discs that were floated on water, a similar decrease in the fluorescence transients were observed, with the lowest degree of reduction being on the second day of the treatment, comprising 20% of the F_o_′ increase to F_s_ (black traces in Figure 5b,d,f,h). In all the discs - wt and *lut2* - the presence of AntA was suppressed, additionally, there was a reduction in the level of the PQ pool, while in the presence of OG, the reduction was higher than it was in the discs without the applied inhibitors. After the recovery period, the transients of the wt discs showed restored values of PQ reduction (g), but for the *lut2* discs, the fluorescence transients demonstrated a low degree of reduction in the PQ pool (h).

### 2.4. Alterations of the Abundance of PGR5 and PTOX as a Result of a Low Temperature and High Light Intensity Treatment for Different Periods of Time

**Figure 6:**
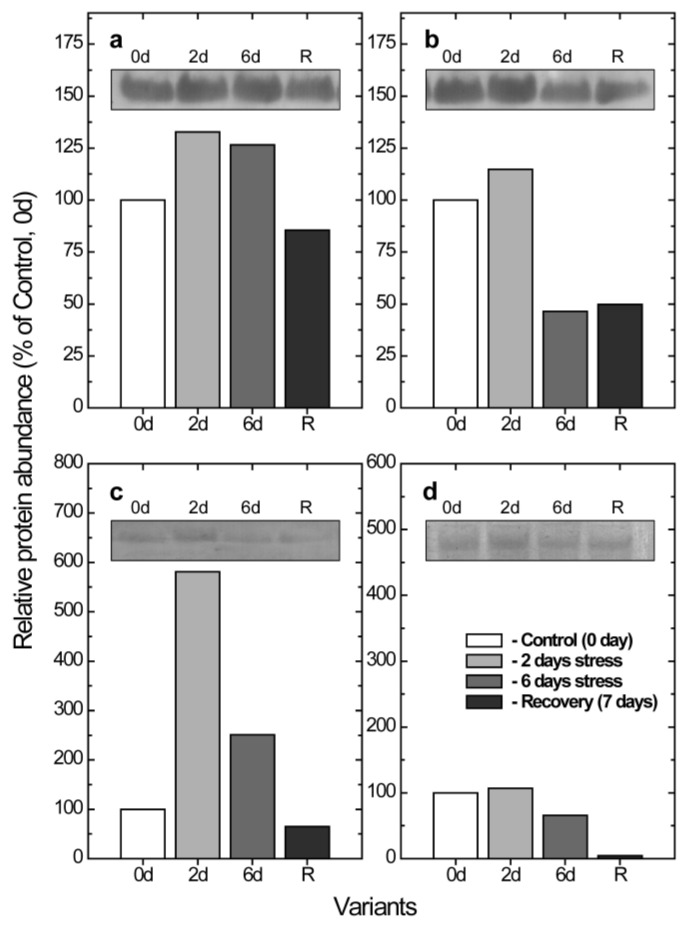
Typical immunoblots (insets) and relative abundance of proton gradient regulation 5 (PGR5) (**a**,**b**) and plastid terminal oxidase (PTOX) (**c**,**d**) polypeptides in thylakoid membranes isolated from wt (**a**,**c**) and *lut2* (**b**,**d**) mutant plants after treatment with high light intensity and low temperature for 2 (2d) and 6 (6d) days, and after recovery for 7 days at control conditions (R). Thylakoid membranes were isolated from leaves of non-treated (0d) and treated plants, separated with sodium dodecyl sulfate–polyacrylamide gel electrophoresis (SDS-PAGE), transferred to polyvinylidene difluoride (PVDF) membrane, and immunodetected with antibodies against PGR5 and PTOX. Presented data are normalized to the relative abundance of the respective polypeptides in the control, non-treated plants (0d).

A quantitative immunoblot analysis of the thylakoid membranes that were probed with antibodies against PGR5 indicated a 132.7% and 126.6% increase of the relative abundance of PGR5 after 2 and 6 days of the combined stress treatment for the wt, respectively, followed by a decline that was close to the values of the control plants after 7 days, during the recovery period, after the stress treatment (Figure 6a).

An increased amount of PGR5 was also registered in the *lut2* plants after 2 days of the treatment, but the effect was much less pronounced (114.8%) when it is compared to that of wt. Interestingly, PGR5 sharply decreased after 6 days of the stress treatment, and it remained low even after the recovery period (Figure 6b). The response of PTOX in the wt, although following the same trend, was much stronger, and the sharp 5.8-fold increase in the PTOX levels was registered after 2 days of the treatment, followed by a significant decrease after 6 days, and PTOX nearly disappeared after 7 days, during the recovery period. (Figure 6c). It should be noted that in the non-treated plants (0d), before the start of the stress treatments, a very low amount of PTOX was registered in the thylakoid membranes of the wt as well as in the *lut2* plants. In contrast to the wt plants, the abundance of PTOX did not change significantly after 2 days of the stress treatment, and it was almost undetectable after 7 days, during the recovery period (Figure 6d).

### 2.5. Chloroplast Ultrastructure in wt and lut2 Mutants

**Figure 7:**
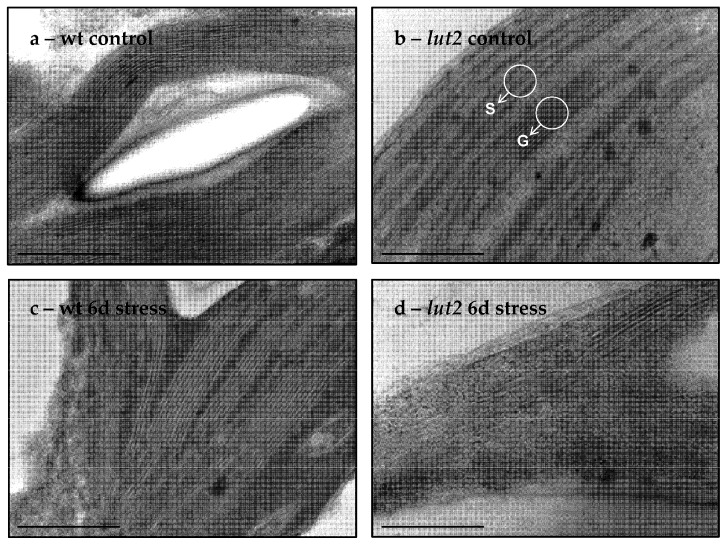
Effect of two stress factors on chloroplast ultrastructure. Typical transmission electron micrographs of mesophyll chloroplasts ultrastructure of wt (**a**,**c**) and *lut2* *Arabidopsis* mutant (**b**,**d**) in control, non-treated plants (**a**,**b**) and after 6 days of treatment with two stress factors (6d stress) (**c**,**d**). The scale bar is equal to 500 nm. Arrows indicate stromal (S) and granal (G) thylakoids.

The transmission electron micrographs indicated typical mesophyll cells of the C3 plants chloroplast ultrastructure, consisting of distinct regions of thylakoids that are organized in grana stacks and lamellar stromal thylakoids in both the non-treated wt (wt control) and the *lut2* (*lut2* control) plants (Figure 7a,b). The exposure of wt *Arabidopsis thaliana* to 6 days of the treatment with two stress factors caused minimal changes in the chloroplast ultrastructure (Figure 7c). In contrast, the exposure of the *lut2* plants to the same stress conditions resulted in a further reduction in the thylakoid granal stacking and mostly single stromal lamellae were present in the *lut2* mutant (Figure 7d) when they are compared to wt under the same stress conditions (Figure 7c). Thus, it appears that the chloroplast ultrastructure in the *lut2* mutant was affected to a much greater extent, upon the treatment with two stress factors than it was in the wt plants.

## 3. Discussion

A comparative investigation [52] of the photosynthetic performance, under the combined treatment of a high light intensity and at a low temperature, of wt of *Arabidopsis thaliana* and lutein-deficient mutant *lut2* has shown that the quantum efficiency of non-photochemical quenching (Φ_NPQ_) and the energy-dependent component q_E_ were lower in the mutants, in comparison with the wt at normal conditions. This could be related to the compromised three-dimensional structure and the organization of LHCII in the absence of lutein [50,53,54,55]. In addition, during development in a high light intensity and a low temperature environment, the wt and *lut2* plants showed a significant increase in the reduced pool of Q_A_ and PQ, which correlated with an enhanced intersystem electron pool size [52,56]. The higher abundance of electrons that can be donated to the PSI reaction center (P_700_^+^) were diverted from the linear electron transport chain and recycled around PSI to provide protection to PSII against photoinhibition. It has to be pointed out that the lutein-deficient *lut2* mutant demonstrated a higher capacity for CEF under the control growth conditions, in comparison with the wt [51,52]. To evaluate the role of alternative electron flows for the response of wt and *lut2* plants to a treatment of high light intensity and low temperature, specific electron transport inhibitors of the major alternative PGR5-dependent pathway (AntA) around PSI [16,57] and octyl gallate (OG), for inhibiting the PTOX-dependent electron transport from PQ pool to molecular oxygen [58,59], were used.

Previous investigations of the *lut2* mutant of *Arabidopsis thaliana* have revealed that the missing lutein in the mutant was replaced by xanthophylls from the brunch of β-carotene synthesis from lycopene [47]. This substitution did not alter the light harvesting functions of the plants, but the abilities of light harvesting monomers to be organized in stable trimers were seriously disturbed [50,53,54,55]. In addition, the 77K fluorescence emission spectra of the thylakoid membranes, isolated from the wt and *lut2* plants and grown at control conditions, indicated that the fluorescence ratio F_735_/F_685_ in the mutant was higher than in the wt [51,60] suggesting that the relative population of PSI complexes in *lut2* was higher than it was in the wild type and/or more energy was delivered to PSI. Taken together, these results imply that in the *lut2* mutant, the stroma-exposed thylakoid membranes, where the PSI complexes are located, prevailed in comparison with those in the wt. Indeed, the presented electron microscopic micrographs clearly demonstrated that the grana structures were less presented in the mutant, while the stroma-exposed thylakoid membranes prevailed in the *lut2*. The applied double stress differentially affected the ultrastructure of the thylakoid membranes. In the wt, the typical structural organization was not significantly altered, while in the *lut2* plants, the population of the stroma-exposed thylakoid membranes in the mutant was further increased. 

Higher plants rely on absorbing sunlight for performing the photosynthetic process. During exposure to abiotic stress conditions, especially at high light intensities and/or low temperatures, an imbalance can occur between the quantity of absorbed light and the temperature-dependent metabolic reactions, leading to the over-reduction of the electron carriers of the electron transport chain, photoinhibition and/or the photo-oxidative stress of PSII and PSI [1,2]. Indeed, the presented data about the photochemical performance of PSII (Φ_PSII_ and q_P_) indicated that both plants, wt and *lut2*, suffered significant stress after the treatment with two stress factors. The negative effect was more strongly expressed and occurred earlier in the mutant. In addition, the performance of PSII was not completely restored in the *lut2* plants upon their return to normal growth conditions. Application of AntA to the wt leaf discs increased the degree of Φ_PSII_ inhibition, indicating that the function of the PGR5-dependent CEF is involved in the protection of PSII from photosynthetic activity under these conditions. The infiltration of leaf discs with AntA resulted in a significant increase in the excitation pressure of PSII in wt, emphasizing the role of the PGR5-dependent CEF for maintaining a more balanced ratio between the excitation and electron flows. In the C3 plants, the PGR5-dependent CEF is the major pathway [29]. Probably, this protection can be also provided by other mechanisms including the NDH-dependent CEF (not a subject of this study) and PTOX-related transfer of electrons from PQ directly to oxygen [43,59]. The PTOX-dependent electron sink is believed to play an important role for the mitigation of the over-reduction of electron transport carriers [43]. The importance of this electron transfer sink in alleviating various environmental stress conditions is supported by the upregulation of PTOX in various plants under light, temperature and salt stresses [11,39,40]. The inhibition of PTOX by OG and the block of this pathway in the wt leaf discs resulted in a relatively small effect on the time-dependence of Φ_PSII_. For the *lut2* plants, the PGR5-dependent CEF does not appear to be so evident as it does in the wt whereas, the electron withdrawal through PTOX affected, more significantly, the response of q_P_ to the high light intensity treatment, which was at a low temperature, especially after 2 days of the treatment. 

The extent of the P_700_ oxidation was negatively affected by the double stress treatment and it occurred earlier in *lut2* in comparison with wt. The application of both inhibitors, AntA and OG, emphasized that the PGR5-dependent CEF and the transfer of electrons from reduced PQ to oxygen by PTOX are important for the response of wt *Arabidopsis thaliana* to treatment with high light intensity, at a low temperature. For the *lut2* plants the participation of the PGR5-dependent CEF in P_700_ oxidation did not seem to be so evident, but the involvement of PTOX was more strongly expressed in comparison with that of the wt. 

The half time of the re-reduction of P_700_ (t_1/2_) in the *lut2* discs from the non-treated plants showed a lower value in comparison with that of the non-treated wt plants, indicating that the rate of CEF through PSI was operating faster in the mutant plants, as was shown previously [51,52]. The contribution of PGR5- and PTOX-dependent alternative electron flows did not seem to contribute significantly to the capacity of CEF in the non-treated wt plants. The proper functioning of PTOX was required for the restoration of the CEF rate in those plants which recovered after the stress, both in wt and *lut2*. 

The post-illumination fluorescence increase of F_o_′ was used to evaluate the extent of the dark reduction in the PQ pool [11,61]. The redox state of the PQ pool is of great importance for the acclimation processes to various extreme environmental conditions. The PQ pool is a key component for the electron trafficking in the photosynthetic apparatus, shared among three pathways of electron transfer—the main LEF, CEF around PSI and the chlororespiratory chain—transferring electrons from stromal reductants to molecular oxygen [62]. 

The transient increase in F_o_′ in the non-treated, *lut2* discs indicated that in the mutant discs, the electron pool in the intersystem electron chain was higher than it was in the wt. The alternative PGR5- and PTOX-dependent electron flows realized a small effect on the dark reduction in the PQ pool in the wt discs, while in the *lut2* discs, the role of both alternative electron flows was much more strongly expressed. The extent of the PQ pool’s reduction in the *lut2* discs with the blocked transfer of electrons from reduced ferredoxin to PQ (AntA-sensitive pathway) was one third of that for the non-treated and those *lut2* discs which floated on water. The effect of the OG in the dark reduction of the transient increase of F_o_′ was less expressed in comparison with that of the AntA. This is a clear indication that the contribution of the PGR5-dependent CEF to the intersystem electron transport chain was higher than that which was mediated by PTOX. Similar results in respect to diminishing the non-photochemical reduction in the PQ pool after treatment with AntA have been reported for *Arabidopsis thaliana* wt and *crr2-2* and *pgr5* mutants [63] and for control and cold-acclimated *Arabidopsis* plants [11]. With an increase in the treatment time, the reduction state in the PQ pool was decreased for both of the investigated plants, wt and *lut2*, but to different extents; in the *lut2* discs, the decrease in the reduction state occurred earlier than it did in the wt, similar to alterations in the photochemical efficiency of PSII and P_700_ oxidation. The application of the AntA inhibitor decreased the reduction in the PQ pool, and this was stronger for the *lut2* treated plants in comparison with the wt that were under the same conditions. For the wt, the presence of AntA led to a noticeable decline in the reduction state, while the OG increased this degree. This result clearly points out the significant contribution of the PGR5-dependent pathway for the redox state of the PQ pool.

PTOX is involved in chlororespiration, carotenoid synthesis and controlling the chloroplast redox state by transferring excess electrons from a reduced PQ pool directly to the molecular oxygen, thus playing a key role in the O_2_-dependent electron sink [11,64]. It was supposed that PTOX performs a regulatory role in poising the redox state of the intersystem electron carriers in the thylakoid membranes, as well to serve a safety valve by preventing the over-reduction of the electron transport players under high light intensities [65]. The over-expression of PTOX helps to diminish the reduction state of the PQ in conditions during which the Calvin cycle is still not active [62]. In agreement with the previous observations, an immunoblot analysis revealed that the abundance of PTOX in both the wt and *lut2* non-treated plants, as well as in the recovered plants, was very low. This is in accordance with already published data, that under normal conditions the PTOX abundance is low and can account for less than 1% of all photosynthetic proteins [37]. In addition, earlier reports also indicated that the over-expression of PTOX did not provide an increased photoprotection in tobacco [37] and *Arabidopsis* [38] plants at optimal growth conditions. The observed sharp (5.8-fold) increase in the PTOX abundance after 6 days of the stress treatment in the wt *Arabidopsis* plants was followed by its decrease near to the control values in the non-treated plants, and this indicated that the PTOX-mediated electron transfer to molecular oxygen was elevated under the combined action of a low temperature and a high light intensity. A similar significant up-regulation of PTOX was reported under various abiotic stress conditions [39,40,41], and the increased abundance of PTOX in plants that were subjected to low temperatures was suggested to guarantee the higher activity of the FQR(PGR5)-dependent PSI cyclic electron flow when the requirement for the PQ pool oxidization was increased [11]. The dynamics of PTOX abundance in the wt during the stress treatments was mirrored by the dynamics of PGR5 abundance, as well as the capacity for PSI-dependent CEF, thus indicating that both PGR5 and PTOX are involved in the regulation of CEF in response to the double stress treatment. Interestingly, although the capacity for CEF in the *lut2* mutant was higher than it was in the wt, and it followed the same response to stress treatments, the abundance of both PGR5 and PTOX only marginally increased after 2 days of the combined stress treatment and it strongly decreased below the values of the control, non-treated, *lut2* plants after 6 days of the stress treatment and remained lower even after recovery. In addition, inhibiting the PGR5-dependent pathway by AntA did not exhibit significant effects on CEF during the stress treatments and the recovery period in the *lut2* plants. This suggests that the major FQR(PGR5)-dependent CEF pathway [29] might be replaced in the *lut2* mutant plants by an AntA-insensitive pathway [30], and the withdrawal of excess electron relays on other-than-PTOX-mediated electron sink(s). 

## 4. Materials and Methods

### 4.1. Plant Growth Conditions

Plants of *Arabidopsis thaliana*, wt (Col-0) and mutant *lut2*, were grown on soil containing perlite at normal growth conditions—illumination with 100 μmol photons m^−2^ s^−1^ flux density (PFD) at day/night, temperature 20/18°C and humidity of 70%. The photoperiod was 12 h. After growth under these conditions for 3–4 weeks, fully developed plants were treated with low temperature (12/10 °C) and high light intensity (500 μmol photons m^−2^ s^−1^) for 6 days, followed by a period of 7 days at control conditions for the recovery period. For the whole experimental setup—plant development, treatment and recovery—plants were grown in growth chambers (Fytoscope FS130, Photon Systems Instruments, Drasov, Czech Republic). Experiments for evaluation of the photosynthetic performance of PSII and PSI were performed before start of treatment, after 2 and 6 days of application of low temperature and high light intensity and after recovery period of 7 days. Two independent experiments were performed with at least 3 parallel samples at every time point. 

### 4.2. Application of Specific Inhibitors

In order to evaluate the contribution of PGR5- and PTOX-dependent pathways to the photosynthetic response of wt and *lut2* plants after the treatment with two stress factors—low temperature and high light—two different specific inhibitors of electron transport were applied: 5 µM antimycin A (AntA) for PGR5 [33] and 10 µM n-octyl gallate (OG) for PTOX [58,59]. Inhibitors were added from stock solutions. Leaf discs were cut from leaves of wt and *lut2* plants, 3 for every treatment, and were floated either on distilled water (Control), or on water containing the respective inhibitor at indicated concentration. So, prepared samples were gently shaken and illuminated with weak light intensity (70 μmol photons m^−2^ s^−1^) for 2 h before registration of photosynthetic parameters of PSII and PSI

### 4.3. Pulse-Amplitude-Modulated Chlorophyll Fluorescence Levels

To determine the photosynthetic performance of PSII, the main fluorescence parameters were registered in the dark, acclimated for 15 min, and leaf discs of wt or *lut2* plants that were floated for 2 h either on distilled water (Control), or on water containing the respective inhibitor. Leaf discs were cut from leaves of plants that were non-treated, treated for 2 or 6 days and after a recovery period of 7 days. Measurements were performed at room (22 °C) temperature and ambient CO_2_ and O_2_ conditions using PAM 101–103 fluorometer (Heinz Walz GmbH, Effeltrich, Germany), as described previously [52]. The minimal chlorophyll fluorescence level at all open PSII centers (F_o_) was registered at illumination with weak (0.120 μmol photons m^−2^ s^−1^) modulated (1.6 kHz) light. To close all PSII centers and to register the maximal fluorescence in the dark-acclimated state (F_m_), a saturating pulse of white light (3000 μmol photons m^−2^ s^−1^) with a duration of 0.8 s was given. Photosynthetic process was initiated with actinic light (AL) equal to the illumination of plants during growth conditions (100 μmol photons m^−2^ s^−1^). Every minute, a saturating pulse of white light was given to determine maximal fluorescence in light acclimated state (F_m_′). After reaching the steady-state fluorescence level (F_s_), the AL was switched off, and the minimal fluorescence in light acclimated state (F_o_′) was detected. 

The alterations in dark non-photochemical reduction in the PQ pool by stroma reductants and/or by electrons from the intersystem electron pool were determined by registration of the post-illumination increase of transients of chlorophyll fluorescence level, after switching off AL and determination of F_o_′ for 90 s [56,61]. For the better visualization of the F_o_′ post-illumination increase in Control and in the presence of AntA or OG, the transients were normalized from 1 (F_s_) to 0 (F_o_′). 

The following fluorescence parameters of PSII activity were calculated according to van Kooten and Snel (1990) [66]. 

The photochemical efficiency of PSII was determined as Φ_PSII_ = (F_m_′ − F_s_)/F_m_′) [67].

Coefficient of photochemical quenching was determined as q_P_ = (F_m_′ − F_s_)/(F_m_′ − F_o_′).

Excitation pressure on PSII was determined as 1-q_P_ = 1 − ((F_m_′ − F_s_)/(F_m_′ − F_o_′)) [1]. 

The relative proportion of absorbed light and dissipated as heat in the antenna of PSII was calculated as 1-(F_v_′/F_m_′) [68].

The variable fluorescence in light acclimated state (F_v_′) was calculated as F_m_′-F_o_′.

### 4.4. Redox State of P700

The stress-induced alterations in the redox state of P700 were determined as described in Ivanov et al. (1998) [6] on dark-acclimated (for 15 min) leaf discs, floated either on distilled water (Control), or on water with respective inhibitor for 2 h. Discs were cut from leaves of wt and *lut2* plants before the start of every experiment, after treatment for 2 or 6 days with two stress factors and after recovery for 7 days at control conditions. PAM–101/103 modulated fluorometer (Heinz Walz GmbH, Effeltrich, Germany) equipped with ED-800T emitter-detector unit [69] was used. Measurements were carried out at 22 °C and ambient O_2_ and CO_2_ conditions. To induce oxidation of P700, the leaf discs were illuminated using FR light (λmax = 715 nm, 10 W m^−2^, Schott filter RG 715). The redox state of P700 was registered as FR-induced absorbance change around 820 nm (ΔA820). When a steady state of FR-induced oxidation of P_700_^+^ was reached (Figure S1), multiple-turnover (MT, 50 ms) and single-turnover (ST, half peak 14 µs) saturating flashes of white light were applied using XMT–103 and XST–103 (Walz) power/control units, respectively. The apparent intersystem electron pool size, the number of electrons that can be donated to PSI, was determined by the ratio between the area of MT and ST flashes. The ST flash leads to a fast reduction of P_700_^+^ by electron flow from the functional reaction centers of PSII [11,70], and is followed by a re-oxidation to the steady P_700_^+^. Application of MT flash leads to a nearly complete reduction of P_700_^+^ reflecting the donation of electrons, not only from PSII, but as well from stroma reductants [56,61,71].

Detection of the half time (t_1/2_) of the decay kinetics of re-reduction of P_700_^+^ after switching off FR illumination [6,69] was applied to determine of the capacity for PSI-dependent CEF [72,73] and/or reduction of P_700_^+^ by stroma reductants [71] in leaf discs cut from wt or *lut2* plant leaves.

### 4.5. Electron Microscopy

Alterations in chloroplast ultrastructure, as induced by exposure of wt and *lut2* plants for 6 days at low temperature and high light intensity, were registered by using a Transmission Electron Microscope HRTEM JEOL JEM 2100 (JEOL Ltd, Tokyo, Japan). One leaf per plant and two plants per treatment were analyzed. Every leaf was cut into strips using a scalpel and were immediately fixed with 4% glutaraldehyde in 0.2 M cacodylate buffer (pH 7.2) for 1 h. The pre-treated samples were post-fixed for 2 h in 1% osmium tetroxide in 0.2 M Na-cacodylate buffer, dehydrated in a series of graded ethanol up to 100% and embedded in Durcupan (Sigma-Aldrich Chemie GmbH, Taufkirchen, Germany) epoxide resin. Ultrathin sections (100 nm thickness) were sectioned using an ultra-microtome Reichert-Jung (Wien, Austria) and put on copper grids, stained with 1% uranyl acetate in 70% methanol and 0.4% lead citrate in dark. The sections were observed and photographed using an electron microscope at accelerating voltage of 220 kV. For every treatment, between 15 and 25 micrographs were taken.

### 4.6. SDS-PAGE Electrophoresis and Western Blot

The effect of growth at low temperature and high light intensity on PGR5 and PTOX relative abundance was evaluated by sodium dodecyl sulfate–polyacrylamide gel electrophoresis (SDS-PAGE) and immunoblot analysis. For the analysis a Laemmli SDS-PAGE system [74] was used—the polyacrylamide concentrations of the stacking and resolving gels were 4 and 12%, respectively. Urea (4 M) was added to the resolving gel. The samples were incubated with a sample buffer (3:1) in the dark for 1 h at room temperature. Thylakoid membranes that were isolated from wt and *lut2* plants before start of treatment (0-day) and from plants grown under double stress for 2 and 6 days and after 7 days of recovery at control conditions were used [75]. Equal amounts of thylakoid membranes corresponding to 3 µg chlorophyll were loaded in every line. The proteins were transferred from SDS-PAGE to polyvinylidene difluoride (PVDF) membrane and proteins were probed with antibodies for PGR5 (AS16 3985—dilution 1:1000) and PTOX (AS16 3692—dilution 1:3000) (Agrisera, Vännäs, Sweden). Development of the blocked membrane was performed using an Alkaline Phosphatase Conjugate Substrate Kit (Bio-Rad, Hercules, California, USA) using a GAR secondary antibody. Densitometric scanning and analysis of each replicate immunoblot was performed with ImageJ 1.41o densitometry software (Wayne Rosband, National Institute of Health, USA, http://rsb.info.nih.gov accessed on 19 July 2022), as described earlier [76]. The presented data were normalized to the relative abundance of PGR5 or PTOX in the control, non-treated plants (0d).

### 4.7. Statistics

Two independent experiments were performed with at least 3 parallel samples per every experimental timepoint (*n* = 6). Statistically significant differences between the values of wt and lut2 plants in the studied variants were identified using two-way ANOVA followed by a Tukey’s post hoc test for each parameter. Prior to the test, the assumptions for the normality of raw data (using the Shapiro–Wilk test) and the homogeneity of the variances (using Levene’s test) were checked. The homogeneity of variance test was used to verify the parametric distribution of data. Values were considered statistically different with *p* < 0.05 after Fisher’s least significant difference post hoc test by using Origin 9.0 for data analysis and graphing software, version 9 (OriginLab, Northampton, MA, USA).

## 5. Conclusions

The presented results have indicated that the stroma-exposed thylakoids prevailed in the *lut2* in comparison with those in the wt of *Arabidopsis thaliana*. The application of two stress factors—low temperature and high light intensity—negatively affected the photosynthetic performance as it was more strongly expressed on the 6th day of treatment for the wt, while the inhibition in the *lut2* started much earlier (on the second day). For the wt *A. thaliana*, the application of the specific inhibitor AntA that blocks PGR5-dependent alternative electron flow resulted in an increased degree of PSII inhibition and an elevated excitation pressure on PSII. However, the effect of the OG that inhibits PTOX-dependent pathway was not so evident. For the *lut2*, the contribution of the PGR5-dependent pathway to photosynthetic performance was not so pronounced as it was in the wt, but the PTOX-mediated transfer of electrons to O_2_ evidently played a more significant role in the response to the exposure to two stress factors, as concluded from the more pronounced effect of the specific inhibitor, OG. The capacity for performing CEF was higher in the *lut2* in comparison with the wt, and it was accelerated by the stress treatment, being better expressed in the wt. The inhibition of the PGR5-mediated pathway did not affect the kinetics of CEF in both the wt and *lut2* plants. The blocking of PTOX realized a well pronounce effect on the rate of CEF, after the recovery period. The electron pool in the intersystem electron chain (reduction in PQ pool) in the non-treated *lut2* was higher in comparison with that in the wt. The degree of reduction in the PQ pool was strongly affected by the growth rate under a high light intensity and at a low temperature, and it was higher in *lut2*. The blocking of PGR5 and PTOX by specific inhibitors further decreased the reduction in the PQ. The stronger effect that AntA had indicated a higher contribution of the PGR5-dependent pathway in the dark reduction in the PQ. The increased PTOX-mediated electron transfer in the course of treatment with the two stressors was confirmed by the increased abundance of this complex, evaluated by a western blot analysis. We suppose that the major PGR5-dependent CEF pathway in *lut2* plants might be replaced by an AntA-insensitive pathway, and a withdrawal of excess electron relays on other-than-PTOX-mediated electron sink(s).

## Data Availability

Not applicable.

## Supplementary Materials

The following supporting information can be downloaded at: https://www.mdpi.com/article/10.3390/plants11172318/s1, Figure S1. Typical trace of far red (FR)-induced P_700_ transients measured at 820 nm. FR light is inducing oxidation of P_700_ (P_700+_). At the steady state of oxidation, a single-turnover (ST) and multiple-turnover (MT) flashes of white saturating light were applied.
