# Supplementary material for: The Role of Alternative Electron Pathways for Effectiveness of Photosynthetic Performance of Arabidopsis thaliana, Wt and Lut2, under Low Temperature and High Light Intensity
Source: Plants (Basel). 2022 Sep 4;11(17):2318. doi: 10.3390/plants11172318 (PMC9460638; doi:10.3390/plants11172318)
Supplement: Supplementary file 1 [file plants-11-02318-s001.zip › plants-1850205-supplementary.pdf]

The role of alternative electron pathways for effectiveness of photosynthetic performance of *Arabidopsis thaliana*, wt and *lut2*, under low temperature at high light

Antoaneta V. Popova[1], Martin Stefanov[1], Alexander G. Ivanov[1,2], Maya Velitchkova[1]

Supplementary **Figure S1**

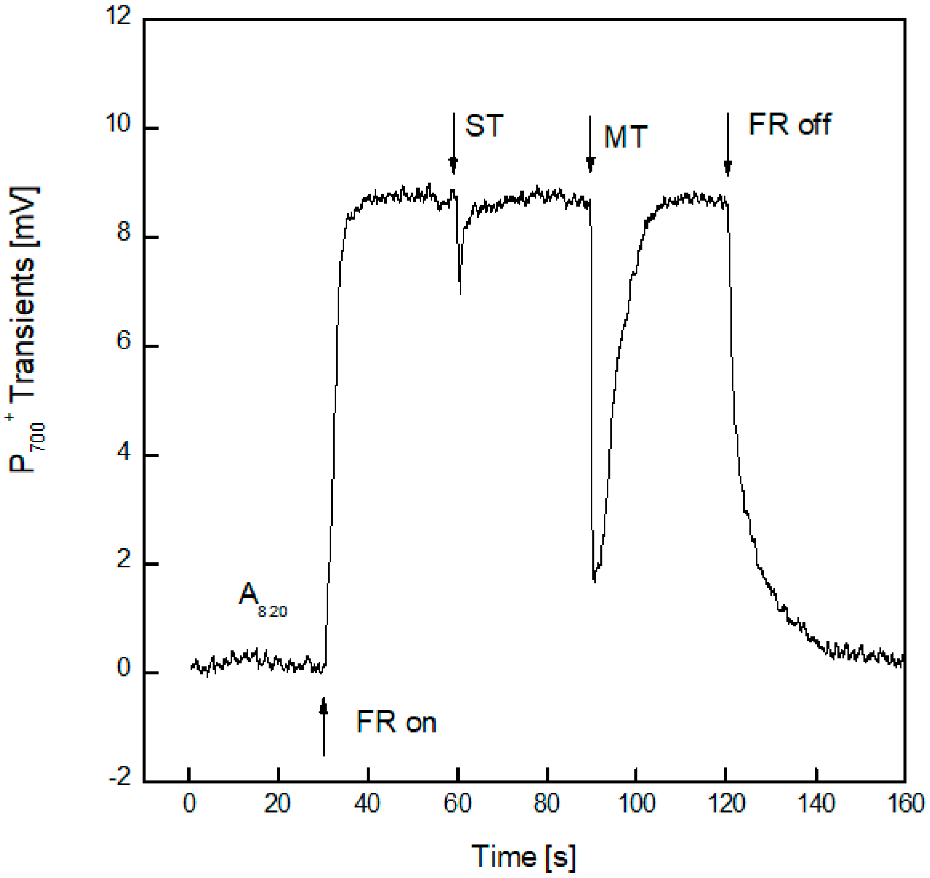

Figure S1. Typical trace of far red (FR)-induced P_{700} transients measured at 820 nm. FR light is inducing oxidation of P_{700} (P_{700}^+). At the steady state of oxidation, a single-turnover (ST) and multiple-turnover (MT) flashes of white saturating light were applied.
